# The spatial heterogeneity of the impact of PM_2.5_ on domestic tourism flows in China

**DOI:** 10.1371/journal.pone.0271302

**Published:** 2022-07-29

**Authors:** Nina Zhu, Ya Luo, Feng Luo, Xue Li, Gang Zeng

**Affiliations:** 1 The Center for Modern Chinese City Studies & Institute of Urban Development, East China Normal University, Shanghai, China; 2 School of Foreign Language, Chengdu College of Arts and Sciences, Chengdu, Sichuan, China; 3 China Institute, Fudan University, Shanghai, China; 4 School of International Economics and Trade, Shanghai Lixin University of Accounting and Finance, Shanghai, China; Northeastern University (Shenyang China), CHINA

## Abstract

As haze pollution intensifies, its impact on tourism is becoming increasingly obvious. However, limited studies have analyzed the impacts of haze pollution on tourism. To explore the contribution rate and impact of PM_2.5_ pollution on tourism flows, panel data on 341 prefecture-level cities in China from 2001 to 2015 were used. The results illustrated that the changes in PM_2.5_ pollution and domestic tourism flows showed a similar partial-most anti-phase main spatial pattern in space, as well as other spatial patterns of PM_2.5_. From a regional perspective, the contribution rate of PM_2.5_ to domestic tourism flows was less than that of traditional factors, such as GDP, GDP_500, and 45A, but larger than that of the Airport factor. The contribution rate of the interaction between PM_2.5_ and 45A on domestic tourism flows was the largest. From a local perspective, PM_2.5_ pollution had a negative impact on domestic tourism flows in northern China, while it had a positive impact in other regions. The classic environmental Kuznets curve (EKC) hypothesis showed applicability to the Chinese tourism industry, and the is of great significance for comprehensively understanding the impact of PM_2.5_ pollution on tourism flows and for promoting the sustainable development of domestic tourism.

## Introduction

With rapid industrialization and urbanization, haze pollution has become one of the most common problems facing the world today. Haze pollution has a serious impact on human health and economic development [1], and in 2014, the United Nations Environment Assembly (UNEA) listed the improvement in air quality as the world’s top priority for sustainable development [2]. As the largest developing country in the world, China has inevitably caused serious pollution problems while pursuing rapid economic development [3–5]. The tourism industry is a pillar for the development of the national economy in China and has entered a stage of qualitative development. However, in recent years, many articles about “haze”, “air pollution”, and “smog”, all of which have negatively impacted the development of China’s tourism industry, have repeatedly appeared in newspapers (see Table 1). The “Annual Report on China’s Inbound Tourism Development in 2015” (http://www.invest-data.com/eWebEditor/uploadfile/2016102009014737247929.pdf), issued by the China Tourism Academy, pointed out that China’s haze pollution has been included in global tourism warnings by international media, which has directly hindered the development of China’s inbound tourism [6, 7]. At the end of 2016, from among 136 countries (regions), China had a lower ranking compared with other countries in terms of clean air, and the sustainability of the tourism environment ranked fifth from the bottom [8]. Therefore, an in-depth analysis of the impact of haze pollution on the development of China’s tourism industry is of great significance for the sustainable development of the tourism economy.

**Table 1:** Newspapers articles on the impact of haze on tourism.

Title	Date	Newspaper
Beijing suffers from heavy pollution, the Palace Museum is shrouded in haze	2021.11.06	China News Service
Cold and haze hit, what made 250 million silver-haired elderly fall in love with winter health tourism?	2020.11.15	Sohu
Escape from smog and get close to nature	2019.11.02	Beijing Travel Network
Travel: Escape from the smog and travel to Ningxia in winter to enjoy the sunshine!	2018.12.08	Weinan Youth Network
What China’s smog means for tourists	2017.06.14	Global Times
Smog spurs Chinese tourism, and tourists seek lung-washing spots	2017.01.11	Fortune Chinese
The rise of green, into a slow city without smog	2016.01.23	Shaanxi Daily
Beijing-Tianjin-Hebei smog "attacks people", and people rush out of the city to "avoid the smog"	2015.12.26	China Securities Journal
Smog has become one of the main influencing factors of my country’s inbound tourism	2014.10.21	Xinhua Daily Telegraph
Air pollution hits Chinese inbound travel hard, hurts Chinese tourism	2013.08.14	Global Times

Scholars have carried out extensive research on the impact of haze pollution on the tourism industry [9–12]. Previous studies have mainly focused on the satisfaction of tourists with destinations experiencing haze pollution, as well as the effect of haze on tourism. For the latter, the impact of haze pollution on inbound tourism has become the main focus, while less attention has been paid to the impact of haze pollution on domestic tourism. Although Xu et al. [13] believed that most domestic tourists paid little attention to haze pollution, the perception of haze, along with the risk perception of inbound tourists, is still weaker than that of domestic tourists; furthermore, inbound tourist loyalty to Chinese destinations is lower than that of domestic tourists. The difference in the perception of haze between domestic and inbound tourists leads to differing effects of haze on the domestic and inbound tourism markets. Thus, investigating the impact of haze on domestic tourism is also very important. In addition, in the research studying the impact of haze on domestic tourism, the overall impact of haze on domestic tourists (income) and its spillover effects are mainly discussed with regard to a certain region, or for all cities in mainland China, from the perspective of spatial correlation [14–16], leaving a lack of discussion on the spatial heterogeneity of the impact of haze on domestic tourists (income).

Due to the vast area of China, the ecological environment conditions, economic development level, population size, and air quality of the different cities varies [17]. At the same time, cities in different locations have varying degrees of tourism resources, as well as different development stages and tourism industry development levels [18]. Therefore, the effect of haze pollution on tourism varies for different cities. Moreover, haze pollution and extreme hot weather will stimulate consumer demand for eco-tourism products to a certain extent, which in turn will force tourism companies to rely on natural tourism resources to provide more eco-tourism products [19–21]. Therefore, destination cities with different types of tourism resources are affected differently by haze pollution. More importantly, for tourist destinations with different degrees of tourism resources, the direction and extent of the impact of haze pollution on the development of their tourism industry may also be different. It is therefore important to analyze the spatial heterogeneity of haze pollution as well as its associated impacts on tourism.

Based on the above results, we investigated the impact of haze pollution on domestic tourism flows (mainly refers to tourism activities carried out within the scope of mainland China) from the perspective of spatial heterogeneity in 341 cities in mainland China from 2001 to 2015. As PM_2.5_ is an important component of haze, this research mainly studies the impact of PM_2.5_ on domestic tourism flows. First, we show the spatiotemporal variances in domestic tourism flows and PM_2.5_, and the spatial relationship between the two from 2001 to 2015. Second, we provide the contribution rate of PM_2.5_ and the interaction effect between PM_2.5_ and other factors on domestic tourism flows from the perspective of spatial heterogeneity. Finally, we explore the spatial heterogeneity of the impact of PM_2.5_ on domestic tourism flows in mainland China.

## Literature review

In recent years, air quality has deteriorated, and haze pollution has frequently been observed all over the world. The serious air quality problems represented by haze pollution have attracted extensive attention from scholars at home and abroad [22]. The research on the relationship between haze pollution and tourism mainly focuses on the following three aspects.

One is the perception of haze by tourists. From the perspective of tourists’ perception, the perception of hazy weather will diminish their willingness to choose a destination [23]. Tourists have a risk perception of haze with regard to physical, functional, psychological, and cost factors, which reduces tourism satisfaction and destination loyalty, while amplifying the negative impact of haze on tourism [23]. In addition, foreign tourists’ awareness, risk perception, and loyalty level regarding China’s haze pollution were lower than that of Chinese tourists. The main reasons that haze pollution has a negative impact on tourism are as follows: Severe weather conditions will increase a tourist’s perception of risks in future travel, and the possibility of potential tourists traveling to the destination will be reduced because of this increase in risk perception [24]. At the same time, haze pollution often makes tourists feel irritable when traveling, reducing their comfort and satisfaction [25]. As a result, the adverse effects of haze pollution on the tourism experience have not only damaged the image of destinations, but have also led to a shrinking of the potential tourism market and a loss of tourism revenue.

The second aspect is the impact of haze on the tourism economy. Relevant studies have found that for every 1% increase in the number of days with air quality below grade II, the proportion of inbound tourism in the GDP drops by 0.309%. PM_2.5_, SO2, smoke, and dust all have a negative impact on the number of inbound tourists and have regional heterogeneity [26]. The study by Day et al. [27] pointed out that weather was an important component of tourist destinations, affecting the demand for tourist destinations, the provision of destination services, the image of the destination, and the economic development of the destination. Hao et al. [28] stated out that differing levels of haze pollution had caused varying degrees of losses to China’s tourism industry. Xu et al. [29] quantitatively explored the heterogeneity of the direct and spillover effects of haze on inbound tourism, and the results showed that those direct and spillover effects were –0.052 and –0.189, respectively. Sajjad et al. [9] regarded deforestation and the depletion of natural resources as two negative indicators of tourism development, and revealed that, on average, haze pollution in Asia and Africa has a negative impact on tourism.

The third aspect is the impact of haze on tourism flows. From the perspective of spatial spillover effects, haze pollution in neighbouring regions will have an impact on local inbound tourism. From 1998 to 2016, haze pollution hindered the balanced development of the inbound tourism industry, causing a significant spatial dislocation between the two [13]. Becken et al. [19] surveyed 600 Australian and US residents and found that air pollution can negatively impact China’s image, thereby reducing the intention of overseas tourists to visit China. However, although many scholars found that haze pollution has a significant negative impact on tourism flows, some contradictory opinions have pointed out that haze pollution has not essentially hindered tourism flows [15].

Overall, existing studies have conducted extensive research on the impact of haze pollution on tourism, but there are still some shortcomings, especially for China. First, most previous studies have focused on the impact of haze pollution on inbound tourism, while the impact of haze pollution on domestic tourism in China is relatively unknown. Second, although the differences in urban characteristics and the composition of pollutants have been agreed upon, studies analyzing the spatial heterogeneity of the impact of haze pollution on domestic tourism are lacking. Third, the impact of haze pollution on tourism flows is still controversial and needs to be analyzed in depth.

## Methodology and data

To investigate the impact of haze pollution on domestic tourism flows in mainland China, various methods were used. First, we showed the spatiotemporal variances of domestic tourism flows and PM_2.5_ from 2001 to 2015 using the empirical orthogonal function method (EOF) method and global Moran’s *I* value. Second, we determined the contribution rate of PM_2.5_ and the interaction effect between PM_2.5_ and other factors on domestic tourism flows using the GeogDetector method. Finally, we explored the spatial heterogeneity of the impact of PM_2.5_ on domestic tourism flows in mainland China using Geographically and Temporally Weighted Regression (GTWR).

### Empirical orthogonal function

The empirical orthogonal function method is used to analyze the main spatial pattern of tourist flow and PM_2.5_ for the past 15 years. The characteristics of spatial change are the most basic characteristics of geographic elements or geographic phenomena and are important in understanding the development law and direction of things [30]. EOF is mainly used to extract the characteristics of the main data. It can describe the original variable field with a small number of spatial distribution modes and can cover a large amount of information on the original variable field [31]. The method has been widely used in the field of meteorology. Its analysis principle is as follows:

Supposing there is a spatiotemporal matrix data whose spatial dimension is *m* and time dimension is *n*, then the matrix can be expressed as follows:

$$X_{ij}=[\begin{array}{ccc} x_{11} & x_{12}\cdots & x_{1n}\\ \vdots & \ddots & \vdots \\ x_{m1} & x_{m2}\cdots & x_{mn} \end{array}](i=1,2,\ldots ,m;j=1,2,\ldots ,n)$$ (1)

Then, the EOF method is to decompose the above matrix into the form of multiplication of the space and time functions, as shown in the following equation:

$$X=VT=\sum_{k=1}^{p}v_{ik}t_{kj},\;i=1,2,\ldots ,m;j=1,2,\ldots ,n$$ (2)

Among them, *V* represents the eigenvector matrix, while *T* represents the time vector matrix. According to orthogonality, the above equation must satisfy the following conditions:

$$\{\begin{array}{c} \sum_{i=1}^{m}v_{ik}v_{il}=1\;k=l\\ \sum_{i=1}^{m}v_{ik}v_{il}=0\;k\neq l \end{array}$$ (3)

Next, we can obtain the eigenvectors, eigenvalues, and time coefficients. By arranging the eigenvalues in descending order (λ_1≥λ_2≥⋯≥λ_m≥0), the variance contribution rate *R*_*k*_ of each eigenvector can be obtained:

$$R_{k}=\frac{\lambda_{k}}{\sum_{i=1}^{m}\lambda_{i}},\;k=1,2,\ldots ,p(p<m)$$ (4)

### Global Moran’s I

The first law of geography states that a correlation is present between things—the closer the distance, the stronger the correlation, and the farther the distance, the weaker the correlation. The main methods for testing a spatial correlation are Moran’s *I*, *G* statistical test, Geary coefficient, LR (likelihood ratio) test, Wald test, spatial error Lagrangian multiplier (LMerr), and spatial lag Lagrangian multiplier (LMlag) [32]. Among these methods, the most popular among scholars is Moran’s *I*, which is divided into global Moran’s *I* and local Moran’s *I* to measure the similarity of the attribute values of an object in adjacent regions of space [33]. The equation of global Moran’s *I* is as follows:

$$I=\frac{n\sum_{i=1}^{n}\sum_{j\neq i}^{n}W_{ij}(x_{i}-\bar{x})(x_{j}-\bar{x})}{\sum_{i=1}^{n}\sum_{j\neq i}^{n}W_{ij}{\left(x_{i}-\bar{x}\right)}^{2}}=\frac{n\sum_{i=1}^{n}\sum_{j\neq i}^{n}W_{ij}(x_{i}-\bar{x})(x_{j}-\bar{x})}{S^{2}\sum_{i=1}^{n}\sum_{j\neq i}^{n}W_{ij}}$$ (5)

Where *I* denotes the Moran index: $S^{2}=\frac{1}{n}\sum_{i}{\left(x_{i}-\bar{x}\right)}^{2},\;\bar{x}=\frac{1}{n}\sum_{i=1}^{n}x_{i}$. The value of Moran’s *I* fluctuates within the range of [−1,1]. A value greater than 0 indicates a positive correlation; less than 0, a negative correlation; and equal to 0, no correlation. The closer its value is to ±1, the stronger the correlation.

The equation of local Moran’s *I* is as follows:

$$I_{i}=\frac{\left(x_{i}-\bar{x}\right)}{S^{2}}\sum_{j\neq i}^{n}W_{ij}(x_{j}-\bar{x})$$ (6)

Where $S^{2}=\frac{1}{n}\sum_{i}{\left(x_{i}-\bar{x}\right)}^{2};\;\bar{x}=\frac{1}{n}\sum_{i=1}^{n}x_{i}$.

### Geogdetector

Spatial heterogeneity is one of the basic characteristics of geographic phenomena [30]. Geogdetector is a tool for detecting and exploiting spatial heterogeneity and revealing forces driving the variability [34]. The advantages of this method are not only its ability to detect both quantitative and qualitative data but its ability to detect the interaction between the two factors [31]. Geogdetector includes 4 detectors: a risk detector, a factor detector, an interaction detector, and an ecological detector [31]. For our research purpose, only the first two detectors are introduced here.

Factor detector: detects the spatial heterogeneity of *Y* and how much a certain factor *X* explains the spatial heterogeneity of *Y*. It is generally measured by the *q* value. The expression is as follows:

$$q=1-\frac{\sum_{h=1}^{L}N_{h}\sigma_{h}^{2}}{N\sigma^{2}}=1-\frac{SSW}{SST}$$ (7)

$$SSW=\sum_{h=1}^{L}N_{h}\sigma_{h}^{2},\;SST=N\sigma^{2}$$ (8)

Where *h* = 1,…, L is the strata of variable *Y* or factor *X*, that is, classification or partition; *N*_*h*_ and *N* are the number of units in layer *h* and the whole area, respectively; and $\sigma_{h}^{2}$ and *σ*^2^ are the variances of the *Y* value of layer *h* and the whole area, respectively. *SSW* and *SST* are within the sum of the square and total sum of the square, respectively. The value range of *q* is [0, 1], where the larger the value, the more obvious the spatial heterogeneity of *Y*. If the strata are generated by the independent variable *X*, then the larger the value of *q*, the stronger the explanatory power of the independent variable *X* on *Y*. In extreme cases, a *q* value of 1 indicates that factor *X* completely controls the spatial distribution of *Y*, while a *q* value of 0 indicates that there is no relationship between factors *X* and *Y*; further, the *q* value indicates that *X* explains 100×*q*% of *Y*.

Interaction detector: identifies the interaction between different risk factors *X*, that is, it assesses whether the combined effects of *X1* and *X2* increase or decrease the explanatory power of the dependent variable *Y*, or whether the effects of these factors on *Y* are independent of each other. The evaluation method is to first calculate the *q* value of both factors *X1* and *X2* to *Y*: *q* (*X1*) and *q* (*X2*), then calculate their interaction *q* value: *q* (*X1*∩*X2*) and compare *q* (*X1*), *q* (*X2*) and *q* (*X1*∩*X2*). The relationship between the two factors can be divided into the following categories (Table 2):

**Table 2:** The types of interactions between the two independent variables and dependent variable [34].

Type	Interaction
*q*(*X1*∩*X2*) < Min(*q*(*X1*), *q*(*X2*))	Nonlinear reduction
Min(*q*(*X1*), *q*(*X2*)) < *q*(*X1*∩*X2*) < Max(*q*(*X1*), *q*(*X2*))	Single-factor nonlinearity reduction
*q*(*X1*∩*X2*) > Max(*q*(*X1*), *q*(*X2*))	Two-factor enhancement
*q*(*X1*∩*X2*) = *q*(*X1*) + *q*(*X2*)	Independent
*q*(*X1*∩*X2*) > *q*(*X1*) + *q*(*X2*)	Nonlinear enhancement

The Geogdetector software is used to calculate Geogdetector. First, we need to calculate the annual average domestic tourism flows, PM_2.5_, GDP, People_500, Traffic, and 45A during the period of 2001 to 2015. Then, these variables corresponding to the 341 cities are divided into five categories according to the natural breaks (Jenks) method by using ArcGIS 10.5 software, respectively. Finally, the processed data is imported into the Geogdetector software for calculation.

### Geographically and temporally weighted regression

Geographically and temporally weighted regression is used to explore the spatial heterogeneity of the influence of haze pollution on tourist flows. The GTWR model can generate a continuous parameter surface by integrating spatial and temporal information into a weighting matrix to capture spatiotemporal variations in local effects [35–37]. Therefore, the spatial heterogeneity of the impact of haze on tourism flows can be effectively identified. GTWR is a spatial analysis method that effectively detects non-stationary features of time and space.


$$y_{i}=\beta_{0}(\mu_{i},\nu_{i},t_{i})+\sum_{k}\beta_{k}(\mu_{i},\nu_{i},t_{i})x_{ik}+\varepsilon_{i},i=1,2,\ldots ,n$$ (9)

Where *i* represents the *i*^th^ observation point; (*μ*_*i*_, *v*_*i*_, *t*_*i*_) represents the coordinates and time of the regression point *i*; *y*_*i*_ represents the dependent variable of point *i*; *x*_*ik*_ represents the *k*^th^ independent variable at the point *i*; *β*_0_ and *β*_*k*_ represent the regression coefficients; *ε*_*i*_ is an independent and identically distributed error term, and it obeys normal distribution. It is usually assumed that the mean is 0 and the variance is σ^2^. Formula (5) is abbreviated to Formula (6).

y=X·β+ε (10)

Where $y=\left[\begin{array}{c} y_{1}\\ y_{2}\\ \vdots \\ y_{n} \end{array}\right]$; X = $\left[\begin{array}{ccc} 1 & \cdots & x_{1k}\\ \vdots & \ddots & \vdots \\ 1 & \cdots & x_{nk} \end{array}\right];\;\varepsilon =\left[\begin{array}{c} \varepsilon_{1}\\ \varepsilon_{2}\\ \vdots \\ \varepsilon_{n} \end{array}\right]$; ‘·’ is the matrix dot multiplication symbol; y, X, ε represents the matrix form of the dependent variable, independent variable, and error term, respectively. The regression coefficient *β* is estimated according to the following Formula (11).


$$\min\sum_{j=1}^{n}W{\left(\mu_{i},\nu_{i},t_{i})(y_{i}-\beta_{0}(\mu_{i},\nu_{i},t_{i})-\sum_{k=1}^{p}\beta_{jk}x_{jk}\right)}^{2}$$ (11)

Where *W*(*μ*_*i*_, *v*_*i*_, *t*_*i*_) is the spatiotemporal weight matrix, which represents the weight matrix of the influence of the regression point *i* on the other observation point *j*, which is solved by fixed and adaptive weight function. The closer the observation value is to regression point *I*, the greater the weight.

According to the least square method, the estimated value of the regression coefficient of the *i*^th^ regression point is shown in Eq (8).


$$\hat{\beta }(\mu_{i},\nu_{i},t_{i})={\left[X^{T}W(\mu_{i},\nu_{i},t_{i})X\right]}^{-1}X^{T}W(\mu_{i},\nu_{i},t_{i})y$$ (12)

Where $\hat{\beta }(\mu_{i},\nu_{i},t_{i})$ is the estimated value the parameter of the *i*^th^ regression point; and *X*^*T*^ represents the transpose matrix of the independent variables.

The fitted value $\hat{y_{i}}$ of the dependent variable at the *i*^th^ regression point is shown in Formula (9).


$$\hat{y_{i}}=x_{i}\hat{\beta }(\mu_{i},\nu_{i},t_{i})=x_{i}{\left[W(\mu_{i},\nu_{i},t_{i})X\right]}^{-1}X^{T}W(\mu_{i},\nu_{i},t_{i})y$$ (13)

Where *x*_*i*_ = (1, *x*_*i*1_, *x*_*i*2_,…,*x*_*ik*_) represents the *i*^th^ row vector in the matrix of independent variable x.

GTWR method was implemented by Matlab 2018a software.

### Variables and data

The explained variable *Y*, domestic tourism flows, is measured as the number of domestic tourists in China, referring to the number of domestic tourist arrivals to the city *i* at year *t* (in 10,000 person-times). The data of domestic tourists in China is taken from the *China Regional Economic Statistical Yearbook* from 2001 to 2015. The explanatory variable is the density of PM_2.5_ (in μg/m^3^), which is taken from NASA’s Global Annual PM_2.5_ Grids data (https://sedac.ciesin.columbia.edu), with a spatial resolution of 0.01° × 0.01°. We use PM_2.5_ to represent haze pollution for three reasons. First, the fine particulate is ubiquitous and still rampant across China. In 2018, 190 out of 338 monitored cities continued to fail to meet the annual standard of 35 μg/m^3^ set by the Chinese government, and none of them met the 10 μg/m^3^ guideline set by the WHO (World Health Organization). Second, PM_2.5_ is a primary ambient pollution with major health risks. It can penetrate deep into the lungs and carry toxins to other organs. High levels of PM_2.5_ irritate respiratory and cardiovascular systems, leading to aggravated asthma, lung disease, and heart attacks [38]. Even a mild level of PM_2.5_ is known to reduce labor productivity and trigger short-term anxiety and depression [39]. Finally, PM_2.5_ has received growing attention in China due to the nascent environmental concerns. A greater awareness of PM_2.5_ implies that more people would have to take costly defensive measures to avoid excess exposure [40]. We use ArcGIS and Python to match each year’s raster data to each city.

The factors that influence tourist flows can be considered from the perspectives of supply and demand [18]. However, in terms of tourism supply, due to the combined effects of various uncertain factors, such as marketing and productivity in tourism supply, reaching a consensus on the characterization indicators of such factors is difficult, making the feasibility of the calculations low. The factors involved in tourist flows are more explicit and direct. Accordingly, we chose the control variables of tourist flows from the perspective of tourism demand to ensure the robustness of the model, and they were at the levels of economic development, market size, local traffic accessibility, and basic tourist attraction of the region [41, 42]. The data was primarily obtained from the *China Regional Economic Statistical Yearbook* from 2001 to 2015, *China Statistical Yearbook* from 2001 to 2015, and the China Scenic Spot website (http://www.chinataa.org) in 2015. The variables are specified below (Table 3):

**Table 3:** Summary of variables.

Variable	Code	Name	Description
**Explained variable**	Y	Tourist	Number of domestic tourists arrivals to a city (ten thousand)
**Explanatory variables**	X1	PM_2.5_	The density of PM_2.5_ (μg/m^3^)
**Control variables**	X2	GDP	Total gross domestic product (billion)
	X3	People_500	Sum of the total people within 500 km
	X4	Traffic	Sum of the number of airport and high-speed rail
	X5	45A	Number of 45A scenic spots

Note: 45A is the sum of AAAA and AAAAA scenic spots, and a 5A scenic area is equal to 2.5 4A scenic areas [43].

• X1 represents the level of haze pollution.

• X2 represents the level of economic development in each region.

• X3 represents the market size in a region. The 500 km here refers to a range within 500 km of a city. With regard to tourism, Wu et al. [44] found that the radius of “transit travel” usually does not exceed 250 km; that is, the distance between two tourist destinations combined for the same source market does not exceed 500 km. Subsequently, Li and Wang further found that this distance is generally not more than 600 km in an effective tour [43]. Considering that there is a large gap in the traffic conditions and residents’ income between the central and western regions of China and the eastern regions, the scope of an effective tour within 500 km is selected here. In addition, the core explanatory variable of this study is PM_2.5_; therefore, no further analysis is made on the scope of the market size. Taking the city as the center, its spherical distance to surrounding cities is calculated. The threshold is set at 500 km, and the total population of the cities within 500 km of the city is determined as the market size of the city.

• X4 represents the local traffic accessibility of each prefecture-level city, as well as the strength of the surrounding area and the local traffic connections. The more convenient the traffic accessibility, the more attractive it is to tourists [45–48]. The traffic is the sum of the number of airports and high-speed rails; that is, if the city has an airport and a high-speed railway station, the traffic of the city is recorded as 2, and if the city has only one airport and no high-speed railway station, the traffic of the city is recorded as 1, and so on.

• X5 represents the basic tourist attractions of a region, being the most popular place for tourists to travel. The number and popularity of tourist attractions in a region strongly influence the development of local tourism.

This study period is from 2001 to 2015. To ensure the comparability of the data, to eliminate their heteroscedasticity, and to ensure stability, all variables were normalized and logarithmized. Therefore, the estimated coefficients of the different variables can be interpreted as elasticities [18]. For some areas, if the relevant data could not be found, we used the growth rate of the area over the years to estimate the missing value of the area in a certain year; for example, in 2011, Anhui Province abolished and divided up Chaohu City, placing it under the management of Hefei, Wuhu, and Ma’anshan cities. In this study, the relevant data of Chaohu City were divided into the corresponding regions according to the land area ratio of the three cities for statistical measurement. The research objects are all prefecture-level administrative regions in mainland China, including prefectures, autonomous prefectures, county-level cities, and leagues, which are all regarded as “city”, among which Chongqing, Shanghai, Tianjin, and Beijing were also deemed “cities”. Meanwhile, Hong Kong, Macao, and Taiwan were not considered. Finally, a total of 341 cities were included.

## Results

### Spatiotemporal patterns of domestic tourism flows and PM_2.5_

To explore the main spatial pattern of domestic tourism flows and PM_2.5_ in the past 15 years, the EOF method was used. Table 4 showed the first seven principal components of domestic tourism flows and PM_2.5_ after EOF decomposition, with each principal component passing the North significance test. When selecting the number of main principal components, we used the 80% standard; that was, when the cumulative variance contribution rate of the current principal components reached 80%, these principal components were considered sufficient to represent most of the domestic tourism flows or PM_2.5_ information. It can be seen that the variance contribution rate of the first principal component of domestic tourism flows was 94.976%, and the principal component variance contribution rate dropped rapidly from the second one. Therefore, only the spatial eigenvector field corresponding to the first principal component was selected for analysis. The spatial eigenvector field corresponding to the first principal component was called the first mode (EOF1), and it represented the main spatial pattern. For PM_2.5_, the cumulative variance contribution rate of the first five principal components was over 80%; thus, the first five spatial eigenvector fields corresponding to the first five principal components represented the main PM_2.5_ information. The variance contribution rate of the first principal component was 50.310% and was greater than the others, so the spatial eigenvector field corresponding to the first principal component was the main spatial pattern, while the other four modes represented the secondary spatial patterns of PM_2.5_. To be consistent with the spatial pattern of tourism flows, we only analyzed the first mode of domestic tourism flows and PM_2.5_, along with their corresponding time coefficients.

**Table 4:** The variance contribution rate and the cumulative variance contribution rate of the first 7 principal components of the domestic tourist flows and PM_2.5_.

	Tourist Flows		PM_2.5_	
	Variance contribution rate (%)	Cumulative variance contribution rate (%)	Variance contribution rate (%)	Cumulative variance contribution rate (%)
**1**	94.976	94.976	50.310	50.310
**2**	1.981	96.957	13.086	63.396
**3**	0.800	97.757	10.902	74.298
**4**	0.441	98.198	4.729	79.027
**5**	0.422	98.620	4.071	83.098
**6**	0.295	98.915	3.617	86.715
**7**	0.266	99.181	2.745	89.460

**Fig 1:**
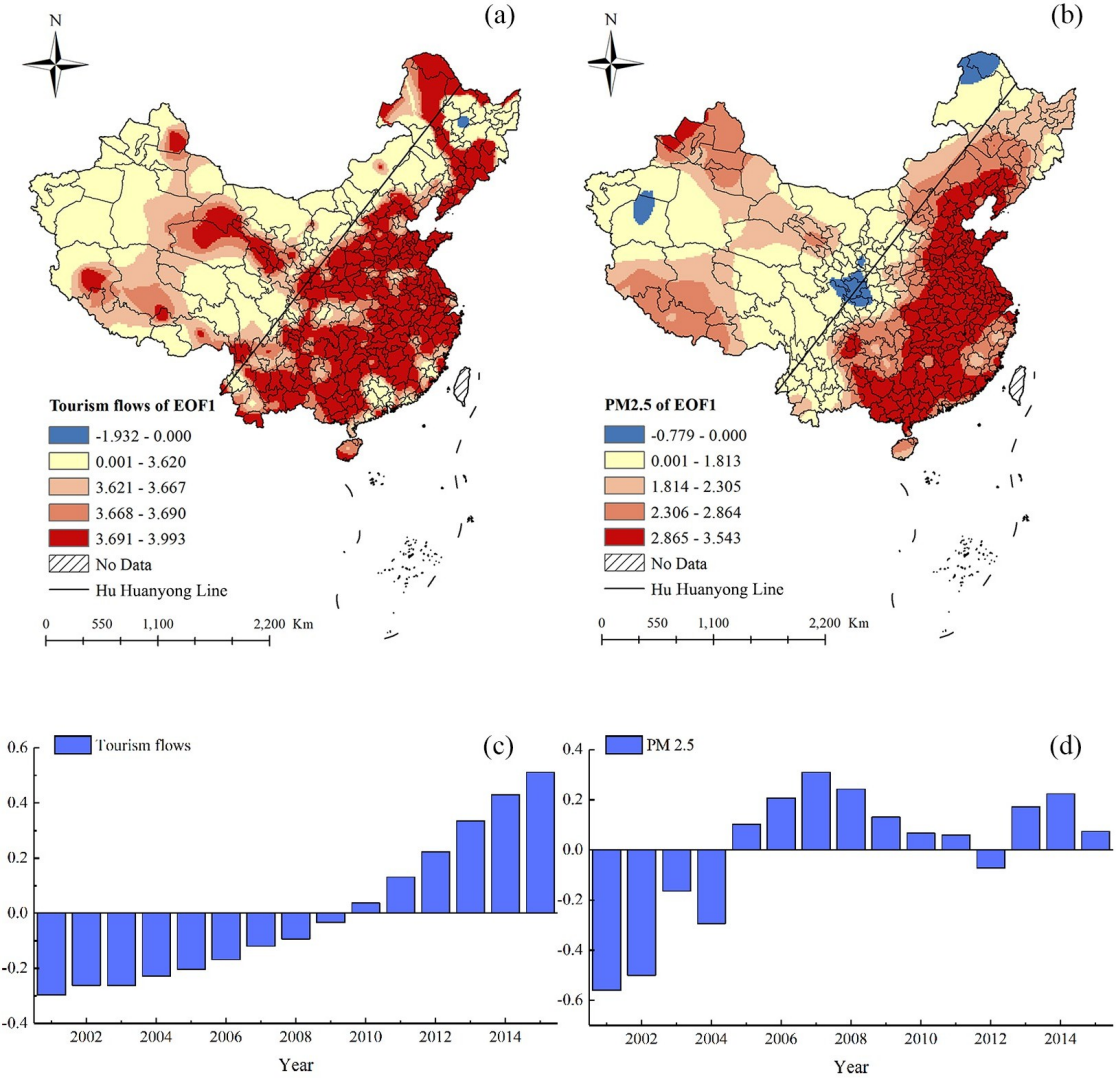
The EOF1 of domestic tourist flows and PM_2.5_ and their time coefficients.

The EOF1 of domestic tourism flows (Fig 1A) represented the main spatial pattern. The magnitude of the value reflected the magnitude of the change in domestic tourism flows. Fig 1A showed that there were both positive and negative values, most of which were positive, while only a small part, namely Suihua City, had negative values, indicating that the changes in domestic tourism flows presented partially mostly anti-phase characteristics. This means that the domestic tourism flows in Suihua City decreased overall, while the domestic tourism flows in other regions had generally increased over the past 15 years. The high-value center of the positive phase was mainly located in the east of the Hu Huanyong Line (HHL), indicating that the domestic tourism flow changes in these areas were relatively large. Meanwhile, the low-value center of the negative phase was located in Suihua City. With regard to the whole country, the changes in the domestic tourism flows in the southeast were much greater than those in the northwest. This may be because the southeast region of the HHL had a high level of economic development, complete tourism infrastructure, prosperous tourism products, and convenient transport [8]. Therefore, it had attracted a large number of tourists in recent years. Fig 1C was the corresponding time series of EOF1 of the domestic tourism flows, and the change in the time coefficient corresponding to the eigenvector represented the change characteristics of the distribution structure represented by the eigenvector. Fig 1C reflected the temporal change in domestic tourism flows from 2001 to 2015, with a negative time coefficient from 2001 to 2009. This indicated that domestic tourism flows in Suihua City decreased during this period. In contrast, the time coefficient was positive from 2010 to 2015, meaning that domestic tourism flows in the rest of the country, increased during this period. The larger the absolute value of the time coefficient, the more obvious the distribution characteristics.

The EOF1 of PM_2.5_ had a similar spatial pattern to domestic tourist flows, and it showed that there were both positive and negative values, most of which are positive, while only a small part, namely Hetian City, Alaer City, Tahe City, some cities in the southeast of Gansu Province, and some cities in the northwest of Heilongjiang Province (Da Hinggan Ling prefecture and Hulunbuir city), had negative values, indicating that the changes in PM_2.5_ presented a partially mostly anti-phase characteristic. This means that the PM_2.5_ level in Hetian City, Alaer City, Tahe City, and some cities in the southeast of Gansu Province decreased overall, while the PM_2.5_ level in other regions increased overall, or vice versa. The high-value center of the positive phase was mainly located in the east of the HHL, indicating that PM_2.5_ changes in those areas were relatively large; conversely, the low-value center of the negative phase was located in Hetian City, Alaer City, Tahe City, and some cities in the southeast of Gansu Province. Similarly, the changes in PM_2.5_ in the southeast were more serious than those in the northwest. The high level of industrialization and urbanization in the southeast of the HHL, its dense population, and high-intensity human activities made the pollution in these areas relatively serious. Fig 1D illustrated the time series of EOF1 of the PM_2.5_ from 2001 to 2015, and the time coefficient was negative from 2001 to 2004 and in 2012, which means that the PM_2.5_ levels in Hetian City, Alaer City, Tahe City, and some cities in the southeast of Gansu Province decreased during this period. Furthermore, the time coefficient was positive from 2005 to 2015, except in 2012, meaning that the PM_2.5_ levels in the whole country except Hetian City, Alaer City, Tahe City, and some cities in the southeast of Gansu Province increased during this period.

### Spatiotemporal correlation of domestic tourism flows and PM_2.5_

**Fig 2:**
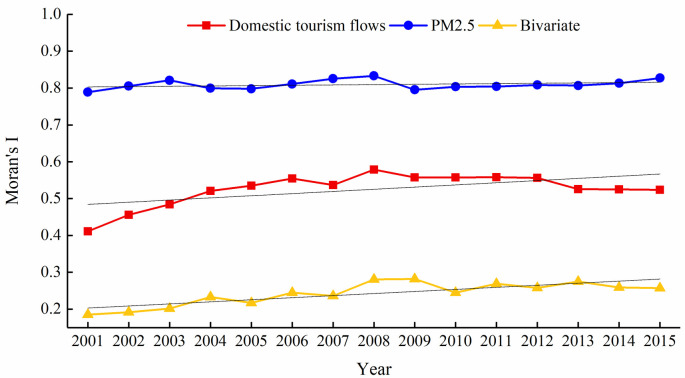
The univariate and bivariate Moran’s *I* of domestic tourism flows and PM_2.5_.

We explored the cluster characteristics of PM_2.5_ and domestic tourism flows and the spatial correlation between PM_2.5_ and domestic tourism flows in 341 cities in China from 2001 to 2015. The specific results are shown in Fig 2.

Domestic tourism flows and PM_2.5_ generally showed strong spatial cluster characteristics. The spatial dependence increased from 2001 to 2015. First, the univariate global Moran’s *I* value of domestic tourism flows and PM_2.5_ were all greater than zero from 2001 to 2015 and passed the 1% level significance test (the *p*-value is omitted in the figure), indicating that the domestic tourism flows and PM_2.5_ had a significant positive spatial correlation; that was, the domestic tourism flows or PM_2.5_ of a region was affected by the domestic tourism flows or PM_2.5_ of the surrounding area. This result regarding domestic tourism flows was consistent with the conclusions of Yang and Fik [18] and those of Mitra et al. [49], thus laying the foundation for the use of the GTWR model below.

Second, it can be seen that the spatial correlation of domestic tourism flows from 2001 to 2015 showed fluctuating changes. Among them, the Moran’s *I* value continued to increase from 2001 to 2008, indicating that the spatial correlation of domestic tourism flows gradually increased. After 2008, the Moran’s *I* value began to show a downward trend, then exhibited slight fluctuations. The reason why the Moran’s *I* value reached its peak in 2008 may be because Beijing hosted the International Olympic Games at this time, which attracted tourists from all over the country to migrate to specific areas, leading to multi-regional cross-border tourist activity. Therefore, the enhanced spatial correlation and cluster of tourism flows resulted in the Moran’s *I* value reach its peak. After the Olympics, the spatial flow of tourism flows began to normalize, thus the Moran’s *I* value began to decline.

Thirdly, the univariate Moran’s *I* value of PM_2.5_ presents a fluctuating upward trend, indicating that the cities with higher annual average PM_2.5_ densities in China tended to be adjacent in space, while cities with low densities were also adjacent in space, and the spatial dependence of PM_2.5_ was increasing. Similar to the Moran’s *I* changes in domestic tourism flows, it continued to increase from 2001 to 2008, then, after reaching a peak in 2008, it showed a slow upward trend.

There is a spatial correlation between PM_2.5_ and the distribution of domestic tourism flows; however, the correlation degree of different years was significantly different, showing an inverted U-shaped change. The bivariate Moran’s *I* of the spatial distribution of PM_2.5_ and domestic tourism flows from 2001 to 2015 was positive, fluctuating between 0.185 and 0.282; furthermore, it showed an inverted U-shaped change and reached a peak in 2009, with significance at the 0.01 level. This result indicated that the spatial correlation of PM_2.5_ and domestic tourism flows was apparent, and the degree of correlation differed over the years.

### The contribution rate of PM_2.5_ to domestic tourism flows

From the above results, we can see that PM_2.5_ was correlated with domestic tourism flows. Compared with some traditional factors, what was the contribution rate of PM_2.5_ to domestic tourism flows? To solve this problem, we chose some traditional factors (the control variables: GDP, People_500, Traffic, 45A) that affected domestic tourism flows to explore the difference between the contribution rate of these factors and PM_2.5_ on domestic tourism flows using the GeogDetector method.

The factor detector was used to detect whether the traditional factors and PM_2.5_ affect domestic tourism flows, and the size of their contribution rates. From Table 5, it could be seen that the *q* value of PM_2.5_, GDP, People_500, Traffic, and 45A were 0.199, 0.542, 0.177, 0.296, and 0.549, respectively, with a corresponding *p*-value for all of 0.000. All passed the 1% level significance test. This means that these traditional factors and PM_2.5_ all had a significant impact on domestic tourism flows, and the contribution rates were 19.9%, 54.2%, 17.7%, 29.6%, and 54.9%, respectively. With the increased of haze pollution and people’s focus on health, tourists had considered the local environmental quality when choosing a destination. Table 5 also indicated that the contribution rate of PM_2.5_ was greater than that of People_500 and close to that of Traffic, which means that the impact of PM_2.5_ on domestic tourism flows played an important role. This result was consistent with that of Xu et al. [15] and Dong [14].

**Table 5:** The result of the factor detector.

	PM_2.5_	GDP	People_500	Traffic	45A
***q* value**	0.199	0.542	0.177	0.296	0.549
***p* value**	0.000	0.000	0.000	0.000	0.000

For the traditional variables (control variables), the contribution rate of 45A was the biggest, followed by GDP, GDP_500, and Airport. Diversified tourism products were the basis for tourists to travel; the more expensive and more famous a region’s tourism products were, the more tourists it would attract. Therefore, 45A was always the most important factor for domestic tourism flows, which was consistent with previous studies [14, 15]. In addition, GDP was also an important factor for domestic tourism flows. In general, GDP represented the level of economic development of a region, and economically developed regions had relatively complete infrastructure, which could meet the diverse needs of tourists and thereby attract more tourists. With the popularization of various modes of transportation and the improvement in its convenience, the impact of transportation conditions on domestic tourism flows was relatively low.

Table 5 indicated that the contribution rate of each factor individually to the domestic tourism flows was different. In fact, if there was an interaction between different influence factors, what was the interaction result? To answer this question, we represented the results of the interaction detector in Table 6.

**Table 6:** The result of the interaction detector.

	PM_2.5_	GDP	People_500	Traffic	45A
**PM**_**2.5**_	0.199				
**GDP**	0.632*	0.542			
**People_500**	0.304*	0.635*	0.177		
**Traffic**	0.527^#^	0.586*	0.474*	0.296	
**45A**	0.667*	0.680*	0.656*	0.625*	0.549

Note

* indicates that the interaction is a bi-enhancement, i.e., *q*(*X1*∩*X2*) > Max(*q*(*X1*), *q*(*X2*))

^#^ indicates that the interaction is a nonlinear enhancement, i.e., *q*(*X1*∩*X2*) > *q*(*X1*) + *q*(*X2*).

Table 5 showed that only the interaction of PM_2.5_ and Traffic had a nonlinear enhancement effect (*q* (PM_2.5_ ∩ Traffic) > *q* (PM_2.5_) + *q* (Traffic)) on domestic tourism flows, while the interactions between the remaining factors had a bi-enhancement effect on domestic tourism flows. This means that the effect of interaction on any two factors is greater than the effect of a single factor. Among them, the interaction effect between GDP and 45A (*q* (GDP ∩ 45A) = 0.680) was the largest, indicating that traditional factors had the most notable effect on domestic tourism flows, whether as a single factor or interaction factors.

It was worth noting that the interaction effect between PM_2.5_ and 45A (*q* (PM_2.5_ ∩ 45A) = 0.667) had the second-largest impact on domestic tourism flows in terms of the interaction between all factors, and had the greatest impact among the results of the interactions dominated by PM_2.5_. Therefore, to better promote the development of tourism, the region should not only focus to the construction of its own tourism facilities, but also work on improving the quality of the environmental. For other control variables, the interaction effect between PM_2.5_ and People_500 (*q* (PM_2.5_ ∩ People_500) = 0.345) was the smallest.

### The spatial heterogeneity of the impact of PM_2.5_ on domestic tourism flows

The above results and previous studies all showed that PM_2.5_ had a significant impact on domestic tourism flows. However, due to China’s vast area, the large differences in geographical environments, and the differing severity of PM_2.5_ in various regions, the impact of PM_2.5_ on domestic tourism flows must be spatially heterogeneous. Table 7 presented the parameter results of the OLS (Ordinary Least Squares), GWR (Geographically Weighted Regression), and GTWR method. It could be seen that the *R*^2^ and Adj*R*^2^ of GTWR were larger than that of OLS and GWR, and the AICc of GTWR was smaller than that of OLS and GWR, indicating that GTWR was the best method for modelling the impact of PM_2.5_ on domestic tourism flows.

**Table 7:** The parameter results of OLS, GWR, and GTWR.

	OLS	GWR	GTWR
***R*** ^**2**^	0.804	0.832	0.871
**Adj*R*** ^**2**^	0.753	0.830	0.871
**AICc**	11120.453	9568.325	8445.371

**Fig 3:**
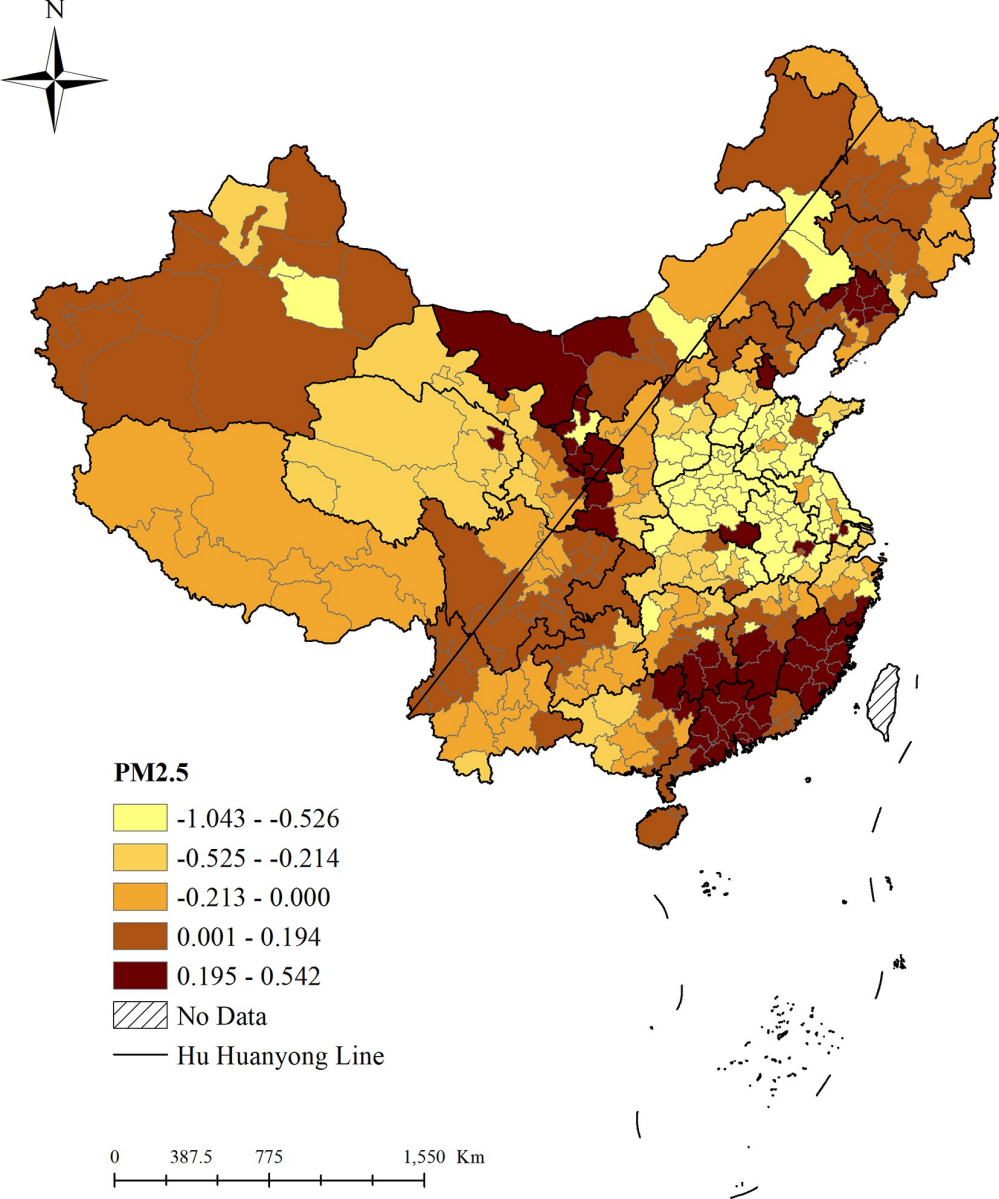
The impact of PM_2.5_ on domestic tourism flows from 2001 to 2015. Note: northeast China includes Heilongjiang Province, Jilin Province, Liaoning Province; southeast China includes Jiangsu Province, Shanghai, Zhejiang Province, Fujian Province, Taiwan, Jiangxi Province, Anhui Province, Guangdong Province, Guangxi Province, and Hainan Province; east China includes Shanghai, Jiangsu Province, Zhejiang Province, Anhui Province, Fujian Province, Jiangxi Province, Shandong Province, and Taiwan; northwest China includes Shanxi Province, Gansu Province, Qinghai Province, Ningxia Hui Autonomous Region, and Xinjiang Uygur Autonomous Region.

Fig 3 showed the results of the impact of PM_2.5_ on domestic tourism flows in mainland China. It could be seen that the impact of PM_2.5_ on domestic tourism flows was spatially heterogeneous; that was, the impact of PM_2.5_ on domestic tourism flows varied in different regions. While the impact was positive in some regions, it could be negative in other regions. Therefore, we not only need to examine the overall impact of PM_2.5_ on domestic tourism flows (for example, Dong et al. (2019) indicated that in mainland China, the overall impact coefficient of PM_2.5_ on domestic tourism flows is -0.007), but also the spatial heterogeneity of the impact of PM_2.5_ on domestic tourism flows.

First, the positive impact of PM_2.5_ on domestic tourism flows was mainly distributed in southeast China, such as the Guangdong Province, Fujian Province, Jiangxi Province, and Hunan Province, as well as some cities in Xinjiang, Inner Mongolia, and northeast China, and this impact was significant (the corresponding *p*-values of regression coefficients passed the 1% significance test). Of these cities, only a few were provincial capitals, while others were ordinary prefecture-level cities. We could see that PM_2.5_ did not hinder the increase in domestic tourism flows in these regions. For every 1% increased in PM_2.5_ in these regions, driven by the current economic growth and strong domestic tourism demand, local domestic tourists would continue to increase by 0.001% to 0.542%. In addition, the areas with the greatest positive impact of PM_2.5_ on domestic tourism flows were mainly located in southeast China. Furthermore, the impact of PM_2.5_ on domestic tourism flows was negative and significant (the corresponding *p*-values of the regression coefficients passed the 1% significance test) in many regions, mainly in the areas of northern China, east China, and central China, as well as Tibet, Qinghai, Gansu, and Yunnan. Among them, the areas with the greatest negative impact of PM_2.5_ on domestic tourism flows were mainly located in east China. Negative impact means that the serious haze pollution led to a decrease in domestic tourists in these regions.

**Fig 4:**
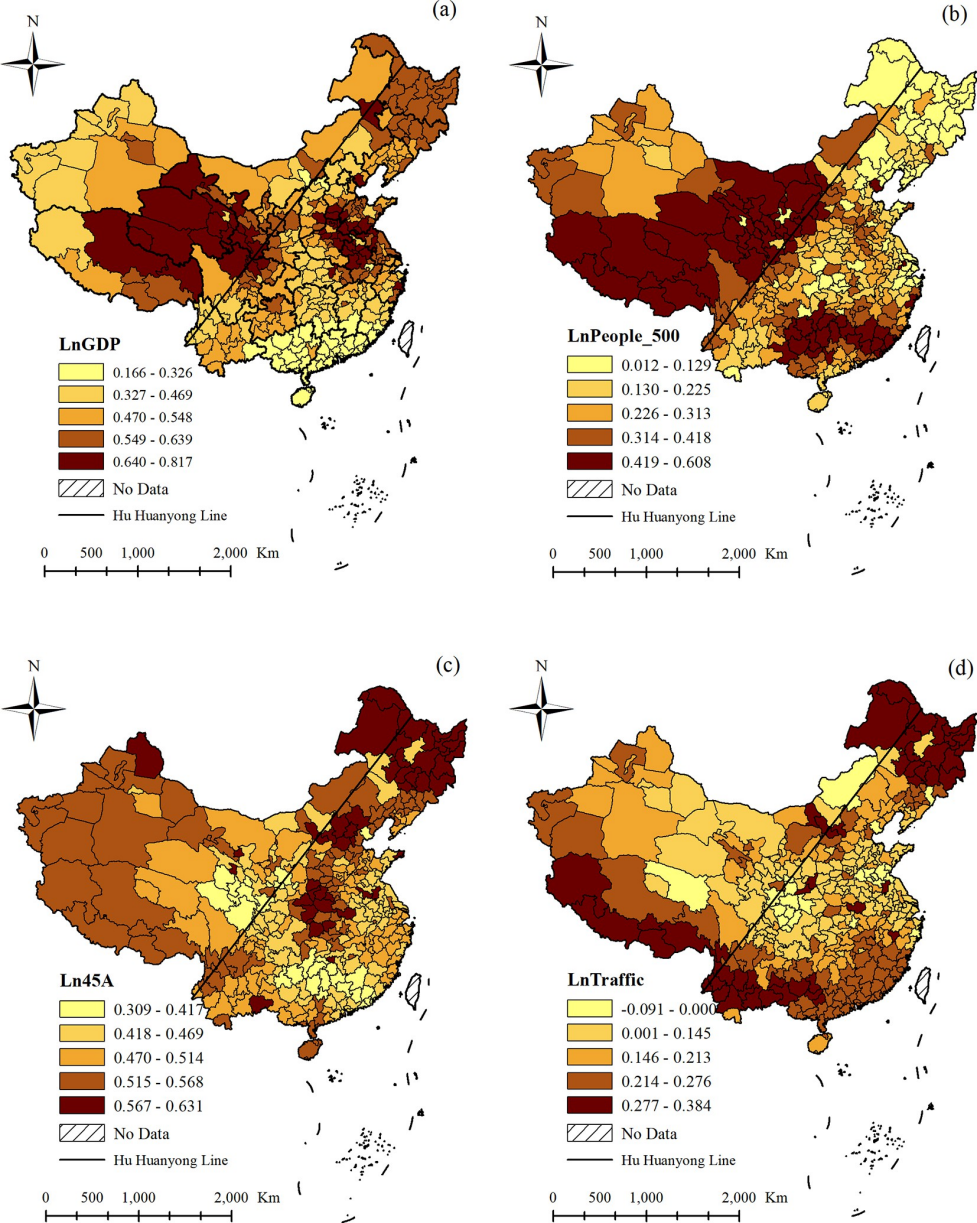
The impact of GDP, People_500, 45A, and Traffic on domestic tourism flows.

For the four control variables, their impact on domestic tourism flows also had spatial heterogeneity. Fig 4(A) showed the impact of GDP on domestic tourism flows. From this figure, we could see that there was a positive and significant impact (the *p*-values of regression coefficients are not showed) for all cities. Among them, the areas that experienced relatively large impacts were mainly in Tibet, Qinghai, Gansu, and some areas in Anhui, Henan, and Shandong. To a certain extent, GDP represented the development level of tourism infrastructure in a region. Since the economic development level was not high in these areas, the tourism infrastructure was not ideal; from Table 4, we can observe that GDP was very important to domestic tourism flows. To attract more tourists, these areas need to vigorously developed their local tourism infrastructure. Fig 4(B) showed the impact of People_500 that represented the effect of the surrounding markets on domestic tourism flows, indicating that the surrounding market also had a positive and significant impact on domestic tourism flows for all cities. This result means that the larger the surrounding market in a region, the greater the number of domestic tourists in that region. Northwest China is particularly rich in tourism resources, but because these regions were sparsely populated and far from the densely populated regions in the east, the surrounding markets had a relatively large impact on the domestic tourism flows in these regions. In addition, the surrounding markets were also important for southeast China, especially in Guangdong and Guangxi. On the contrary, the impact of People_500 on domestic tourism flows in northeast China was smaller. Fig 4(C) showed the impact of 45A that represented the effect of tourism products on domestic tourism flows. There was a positive and significant impact for all cities, which was different from that of GDP and People_500; the areas with the largest impact of 45A on domestic tourism flows were mainly in northeast China. This region was home to Changbai Mountain, Songhua River, and other famous tourism destinations, which attract increasingly more tourists with their unique northern customs; therefore, the impact of 45A on domestic tourism flows in this region was the largest. Fig 4(D) displayed the impact of traffic on domestic tourism flows. Similar to the impact of 45A on domestic tourism flows, the impact of traffic was also larger in northeast China. In addition, the impact of traffic on domestic tourism flows was larger in Tibet, Yunnan, and Guangxi. It could be seen that these cities were all located on the border of China, and were all far from densely populated cities. Thus, to attract more tourists, traffic was very important for these cities.

## Discussion

The purpose of our study was to explore the spatial heterogeneity of the impact of PM_2.5_ on domestic tourism flows in China from 2001 to 2015. To this end, we first analyzed the spatial pattern of PM_2.5_ and domestic tourism flows using the EOF method. The results show that they have a similar partially mostly anti-phase main spatial pattern, and that the changes in the area southeast of the HHL were more sensitive during the study period. In recent years, the domestic tourism industry has developed rapidly, and the number of domestic tourists, on the whole, has continued to increase in China. However, from a spatial perspective, not all regions have seen an increase in domestic tourism flow. On the contrary, the domestic tourism flow in some individual cities has shown a decreasing trend, especially in Suihua City. At the same time, PM_2.5_ levels did not show an increasing trend nationwide, resulting in apparent spatial heterogeneity; thus, we need to analyze the domestic tourism flows and PM_2.5_ from the perspective of spatial heterogeneity. In addition, domestic tourism flows and PM_2.5_ not only have spatial autocorrelation, but there is also a cross-correlation between them. The domestic tourism flows in a city are not only influenced by the surrounding domestic tourism flows, but also by the surrounding PM_2.5_ levels, which once again proved that PM_2.5_ will have an impact on domestic tourism flows. Therefore, in the current study of tourism flow, we must consider the impact of PM_2.5_.

Our results show that the impact of PM_2.5_ on domestic tourism flows is significant from the perspective of spatial heterogeneity, which means that PM_2.5_ is an important factor that should not be ignored by tourists. Our results are consistent with previous studies [14, 15], although those studies discussed the impact of PM_2.5_ on domestic tourism flows from the perspective of spatial correlation. In addition, we are the first to point out that the contribution rate of PM_2.5_ is 19.9%, which is greater than the contribution rate of the surrounding market (People_500). It is clear that the impact of PM_2.5_ on domestic tourism flows is now greater than some of the traditional factors. Regarding the traditional factors that affect tourism flows, GDP and 45A have a large contribution rate, but local economic development and tourism products are still the most effective means to promote the growth of the tourism market. Moreover, improvement in other factors also helps to promote the growth of tourism flows, but there are obvious differences in their intensity of action. Each driving factor had an effect on domestic tourism flows but did not work alone; rather, different driving factors interacted with each other, having an enhanced influence on domestic tourism flows.

Although the impact of PM_2.5_ on domestic tourism flows is positive and significant overall, there is apparent spatial heterogeneity. For the areas in north and east China where the PM_2.5_ pollution is more serious, PM_2.5_ has a significant negative impact on tourism flows. With the increase in haze pollution, those tourists that are more sensitive to the environment will abandon their travel plans due to the poor air quality of the destination [19, 26]. Furthermore, for some tourist destinations that are mainly visual spectacles, such as those that offer water sightseeing and biological landscapes, severe PM_2.5_ pollution will reduce the visual effect and greatly reduce the satisfaction of tourists. Severe PM_2.5_ pollution can even lead to the cancellation of flights or high-speed trains, forcing tourists to cancel their travel plans. All of these will affect domestic tourism flows. In contrast, for the areas where the PM_2.5_ pollution is not very serious, PM_2.5_ has not hindered tourism flows, but rather has promoted it. PM_2.5_ pollution in most cities in China is difficult to tackle in a short time. However, the classic environmental Kuznets curve (EKC) hypothesis has shown applicability to the Chinese tourism industry. For a developing country such as China, tourism flow is an important indicator of economic phenomena [50, 51]. It is unrealistic to ignore the inverted U-shaped relationship between environmental pollution and economic growth in the EKC. The domestic tourism flows show an upward trend, which is in line with the EKC hypothesis. On the other hand, these regions have a better natural environment. Although PM_2.5_ pollution has increased in recent years, it has promoted tourism-related enterprises and encouraged the destination’s governments to update their business concepts and develop new low-pollution tourism products [52]. For example, weather with serious PM_2.5_ pollution has reduced the attractiveness of urban tourism, therefore, rural and eco-tourism have been pursued; weather with serious PM_2.5_ pollution has also promoted the regional tourism industry to develop and operate products in accordance with the ecological concepts in various areas such as food, housing, travel, shopping, and entertainment. Tourism products and routes are focusing more attention on environmental protection, while related companies have launched a new type of insurance called “tourism haze insurance” as a tourism service product to combat the damage and uncertainty caused by haze pollution. These have all promoted an increase in domestic tourism flows.

However, there are some limitations in this study. First, due to a lack of data, the cut-off time for this study is 2015, which means that the impact of PM_2.5_ on tourism in recent years cannot be effectively identified. In future studies, it will be necessary to continuously improve the data to explore the relationship between PM_2.5_ and tourism. Second, both tourism flow and PM_2.5_ have seasonal characteristics. Due to the data availability limitations, this study did not explore the seasonal differences in the impact of PM_2.5_ on tourism flows. These studies need to be strengthened in the future.

## Conclusions

The study first analyzed the spatiotemporal variations in domestic tourism flows and PM_2.5_ pollution in mainland China from 2001 to 2015 using the EOF method. We then investigated the spatiotemporal correlation of domestic tourism flows with PM_2.5_ at different times. Next, the contribution rate of PM_2.5_, the interaction effect of PM_2.5_, and the influence of some traditional factors on domestic tourism flows from 2001 to 2015 were explored using the GeogDetector method. Finally, we assessed the spatial heterogeneity of the impact of PM_2.5_ on domestic tourism flows at different times using the GTWR method. Summarizing this study, the main conclusions are as follows:

1. There are different spatial patterns of domestic tourism flows and PM_2.5_. The main spatial patterns show partially-mostly anti-phase characteristics. Domestic tourism flows and PM_2.5_ show an increasing trend in most areas, while only a few areas show a decreasing trend. In addition, PM_2.5_ also shows four other minor spatial patterns.
2. There is a positive and significant spatial correlation between domestic tourism flows and PM_2.5_ in space. Over time, the relationship between the two has developed an inverted U-shaped characteristic. Therefore, when formulating relevant tourism policies, we must not only consider the local domestic tourism flows, but also the surrounding domestic tourism flows and PM_2.5_.
3. The contribution rate of PM_2.5_ to domestic tourism flows is less than that of GDP, GDP_500, and 45A, and is larger than that of Airport. In addition, there is an interaction effect between different control variables and PM_2.5_; among them, the interaction effect between PM_2.5_ and 45A is the largest.
4. The impact of PM_2.5_ on domestic tourism flows has apparent spatial heterogeneity. The significant and positive impacts of PM_2.5_ on domestic tourism flows are mainly distributed in the southeast of China, such as the Guangdong Province, Fujian Province, Jiangxi Province, and Hunan Province, as well as some cities in Xinjiang, Inner Mongolia, and northeast China. Most of them are ordinary prefecture-level cities. The impact of PM_2.5_ on domestic tourism flows is negative and significant in many regions, mainly distributed in the areas of North, East, and Central China, as well as Tibet, Qinghai, Gansu, and Yunnan. The classic environmental Kuznets curve (EKC) hypothesis has shown applicability to the Chinese tourism industry.
